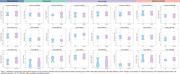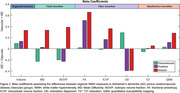# Can neuroimaging methods help us to disentangle WMH etiology in AD?

**DOI:** 10.1002/alz.085994

**Published:** 2025-01-09

**Authors:** Mahsa Dadar, Olivier Parent

**Affiliations:** ^1^ Douglas Mental Health University Institute, Montreal, QC Canada; ^2^ McGill University, Montreal, QC Canada

## Abstract

White matter hyperintensities (WMHs) are frequently observed in ageing individuals, and have a higher prevalence in neurodegenerative disorders such as Alzheimer’s disease. Ex‐vivo assessments of the microstructural alterations within WMHs have reported heterogeneous tissue alterations, with demyelination, axonal loss, and inflammation presenting with various degrees of severity. There is a crucial need to better assess the severity of WMH microstructural alterations in vivo, in particular with the emergence of anti‐amyloid immunotherapies and the associated risk of Amyloid Related Imaging Abnormalities (ARIAs) in individuals with comorbid vascular disease. Recent in‐vivo and ex‐vivo investigations have revealed important aetiology differences in regional WMHs, linking frontal WMHs to more vascular factors (e.g. ischaemia) and posterior WMHs to neurodegenerative processes. Regional WMH features might therefore reveal additional information regarding their potential aetiologies in absence of postmortem neuropathology information. Novel neuroimaging techniques such as diffusion‐weighted imaging (DWI) and quantitative magnetic resonance imaging (qMRI) can also provide further insight into the specific pathophysiological properties of the WMHs. These microstructural MRI metrics allow for the characterization and measurement of iron and myelin density, axonal integrity, and water content, paving the way for deep phenotyping of the pathophysiology of WMHs in‐vivo. Using DWI and qMRI data from the UKBiobank, we assessed the differences in baseline volume, mean diffusivity (MD), isotropic volume fraction (ISOVF, an index of relative extracellular water diffusion), fractional anisotropy (FA), intracellular volume fraction (ICVF, an estimate of neurite density), orientation dispersion (OD, reflecting the directional complexity of diffusion), T2* relaxation, and quantitative susceptibility mapping (QSM, sensitive to myelin and iron) of periventricular, anterior, and posterior WMHs between those with a diagnosis of AD (N=15) and cerebrovascular disease (N=95) in their future visits (mean interval: 3.3years). Our results show differences in their baseline WMH pathophysiological and spatial patterns (Figure 1), with cerebrovascular pathologies having a higher WMH burden on most metrics, especially in anterior regions. Figure 2 shows the standardized beta coefficients of the models, controlling for age and sex. Combined, regional volumetric and signal measures might therefore be able to provide important additional information regarding the aetiology of WMHs at an individual level.